# Unraveling the Rare Association Between Ulcerative Colitis and Posterior Reversible Encephalopathy Syndrome: A Case Report

**DOI:** 10.7759/cureus.76617

**Published:** 2024-12-30

**Authors:** Abhinav Kadam, Jiwan Kinkar, Saket S Toshniwal, Sunil Kumar, Suhit Naseri

**Affiliations:** 1 Department of Medicine, Jawaharlal Nehru Medical College, Datta Meghe Institute of Higher Education and Research, Wardha, IND; 2 Department of Neurology, Jawaharlal Nehru Medical College, Datta Meghe Institute of Higher Education and Research, Wardha, IND; 3 Department of Pathology, Jawaharlal Nehru Medical College, Datta Meghe Institute of Higher Education and Research, Wardha, IND

**Keywords:** epilepsy, inflammatory bowel disease, magnetic resonance imaging, neurological symptoms, pres

## Abstract

Brain edema and neurological symptoms are the hallmarks of the uncommon disease known as posterior reversible encephalopathy syndrome (PRES), which can have several etiological causes. Since the etiology determines the course of treatment, diagnosis is crucial. There have only been 14 cases of PRES associated with inflammatory bowel disorders documented. We present an exciting and novel presentation of a 19-year-old female who was admitted to the hospital with a diagnosis of acute severe colitis and experienced multiple seizure episodes. She was later diagnosed with atypical PRES on an MRI brain, which showed posterior white matter T2 lesions with anterior extension. She was started on anti-epileptic drugs and showed regression of symptoms within 24 hours. The follow-up MRI showed complete resolution. Furthermore, a colonoscopy was done, which was suggestive of ulcerative colitis. The association of ulcerative colitis with atypical PRES is rarely documented in the literature.

## Introduction

The rare disease known as posterior reversible encephalopathy syndrome (PRES) was initially reported by Hinchey et al. [1]. The following characteristics of the clinical and radiologic investigations are present: (i) subcortical vasogenic edema in the white matter of the occipital and/or parietal lobes as demonstrated by MRI; (ii) neurological symptoms, including headache, seizure, altered consciousness, focal neurological deficits, and visual abnormality; and (iii) resolution of symptoms and imaging findings in the majority of cases [1]. Acute blood pressure fluctuations or inflammatory cytokines may produce endothelial dysfunction and increased vascular permeability; however, the exact origin of the condition is unknown [2].

Crohn's disease (CD) and ulcerative colitis (UC) are the two most common chronic inflammatory disorders that are included under the umbrella term of inflammatory bowel disease (IBD). Numerous symptoms, including diarrhea, bloody stools, weight loss, stomach discomfort, fever, and exhaustion, are shared by these two illnesses [3].

IBDs account for 4.4% of patients in big series from the Mayo Clinic in PRES, although it is a rare diagnosis [4]. In adults with IBD, PRES has been linked to azathioprine, infliximab, and cyclosporin [5]. Seldom does PRES manifest in individuals with IBDs in its atypical form. Recognizing PRES early in UC patients is crucial but challenging, as the symptoms might overlap with the side effects of medications used in UC management, such as corticosteroids or immunosuppressants. This makes the case clinically complex and rare in presentation [5].

We report a case of a 19-year-old female presenting with atypical PRES, without any drug association, in a newly diagnosed case of UC.

## Case presentation

A 19-year-old female presented to the hospital in April 2024 with complaints of abdominal pain and loose stools for two days. She stated that abdominal pain was colicky, diffuse, and non-radiating. The loose stools were said to be non-blood stained, and the frequency was approximately 16 episodes. The patient did not have any comorbidity. A suspicion of acute colitis was kept, and she was admitted to the female medicine ward for further management.

General examination revealed that the patient was afebrile; her pulse was 106 beats per minute (bpm), her blood pressure was 100/70 mmHg, and her oxygen saturation was 99% on room air. Severe pallor was present. Rest general examination and systemic examination revealed normal findings. Routine investigations were done (Table 1).

**Table 1:** The biochemical and pathological laboratory investigations of the patient on admission. MCHC, mean corpuscular hemoglobin concentration; MCV, mean corpuscular volume; MCH, mean corpuscular hemoglobin; RBC, red blood cell; WBC, white blood cell; RDW, red cell distribution width; APTT, activated partial thromboplastin time; INR, international normalized ratio; SGOT, serum glutamic oxaloacetic transaminase; SGPT, serum glutamic pyruvic transaminase; HIV, human immunodeficiency virus; ESR, erythrocyte sedimentation rate; CRP, C-reactive protein

Laboratory parameter	Results	Normal values
Hemoglobin	5.4 g/dL	11-14 g/dL
MCHC	34.4 g/dL	32-36 g/dL
MCV	74 micron	79-92 micron
MCH	32.4 pg	27-31 pg
Total RBC count	3.91x10^6 ^cells/cumm	2.50-5.50x10^6 ^cells/cumm
Total WBC count	5600 cells/cumm	4000-11000 cells/cumm
Total platelet count	1.87x10^6 ^cells/cumm	1.50-4.50x10^6 ^cells/cumm
Hematocrit	42.6%	40-54%
Monocyte	03%	2-8%
Granulocyte	56%	40-60%
RDW	17.6 fL	12.2-16.1 fL
Eosinophils	01%	1-4%
Basophil	0%	<1%
APTT	29.8 seconds	29.5 seconds
Prothrombin time	13.2 seconds	11.3 seconds
INR	1.05	1.00
Urea	18 mg/dL	6.24 mg/dL
Creatinine	0.8 mg/dL	0.59-1.04 mg/dL
Sodium	134 mEq/L	135-145 mEq/L
Potassium	3.8 mEq/L	3.5-5.1 mEq/L
Alkaline phosphate	78 IU/L	75-124 IU/L
SGOT	48 IU/L	8-45 IU/L
SGPT	49 IU/L	7-56 IU/L
Total protein	7.2 g/dL	6.0-8.3 g/dL
Albumin	4.7 g/dL	3.4-5.4 g/dL
Total bilirubin	0.6 mg/dL	0.1-1.0 mg/dL
Conjugated bilirubin	0.2 mg/dL	0.1-0.4 mg/dL
Unconjugated bilirubin	0.4 mg/dL	0.2-0.6 mg/dL
HIV card test	Negative	-
Serum amylase	116 U/L	30-120 U/L
Serum lipase	66 U/L	13-78 U/L
ESR	39 mm/hr	0-20 mm/hr
CRP	28 mg/L	<10 mg/L
Random blood sugar	95 mg/dL	90-110 mg/dL

Because of severe anemia, the patient was planned for two units of packed red cells (PRC) transfusion, and further supportive management was initiated. After two hours of admission, the patient started to experience altered sensorium and experienced generalized tonic-clonic seizures (GTCS), which were associated with uprolling of eyes, tongue bite, and drooling saliva followed back by a period of unconsciousness for two episodes. An immediate neurologist's opinion was sought, which recommended an MRI of the brain. After stabilizing the seizure activity, a detailed neurological examination was conducted, revealing normal higher mental functions, a normal cranial nerve examination, and normal motor and sensory functions, with brisk deep tendon reflexes in all four limbs and a bilateral extensor plantar response.

**Figure 1:**
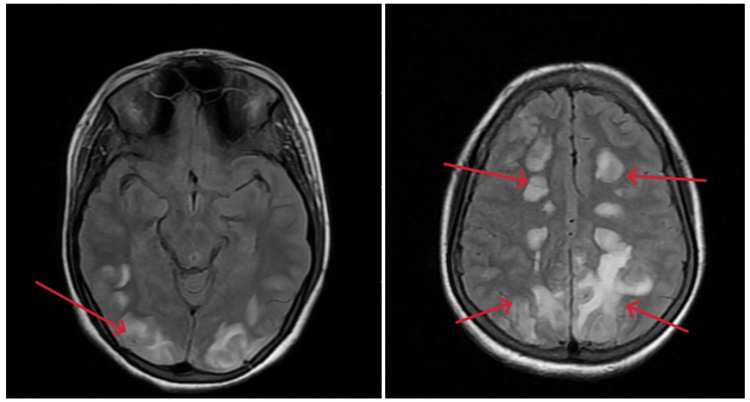
MRI of the brain (axial section) showing bilateral symmetrical altered signal intensity (red arrows) in the subcortical white matter of the occipital and parietal lobes, extending to the frontal lobes, on T2/FLAIR-weighted imaging. FLAIR, fluid-attenuated inversion recovery

MRI of the brain showed bilateral symmetrical altered signal intensity in the subcortical white matter of the occipital and parietal lobes, extending to the frontal lobes, on T2/fluid-attenuated inversion recovery-weighted imaging (Figure 1).

**Figure 2:**
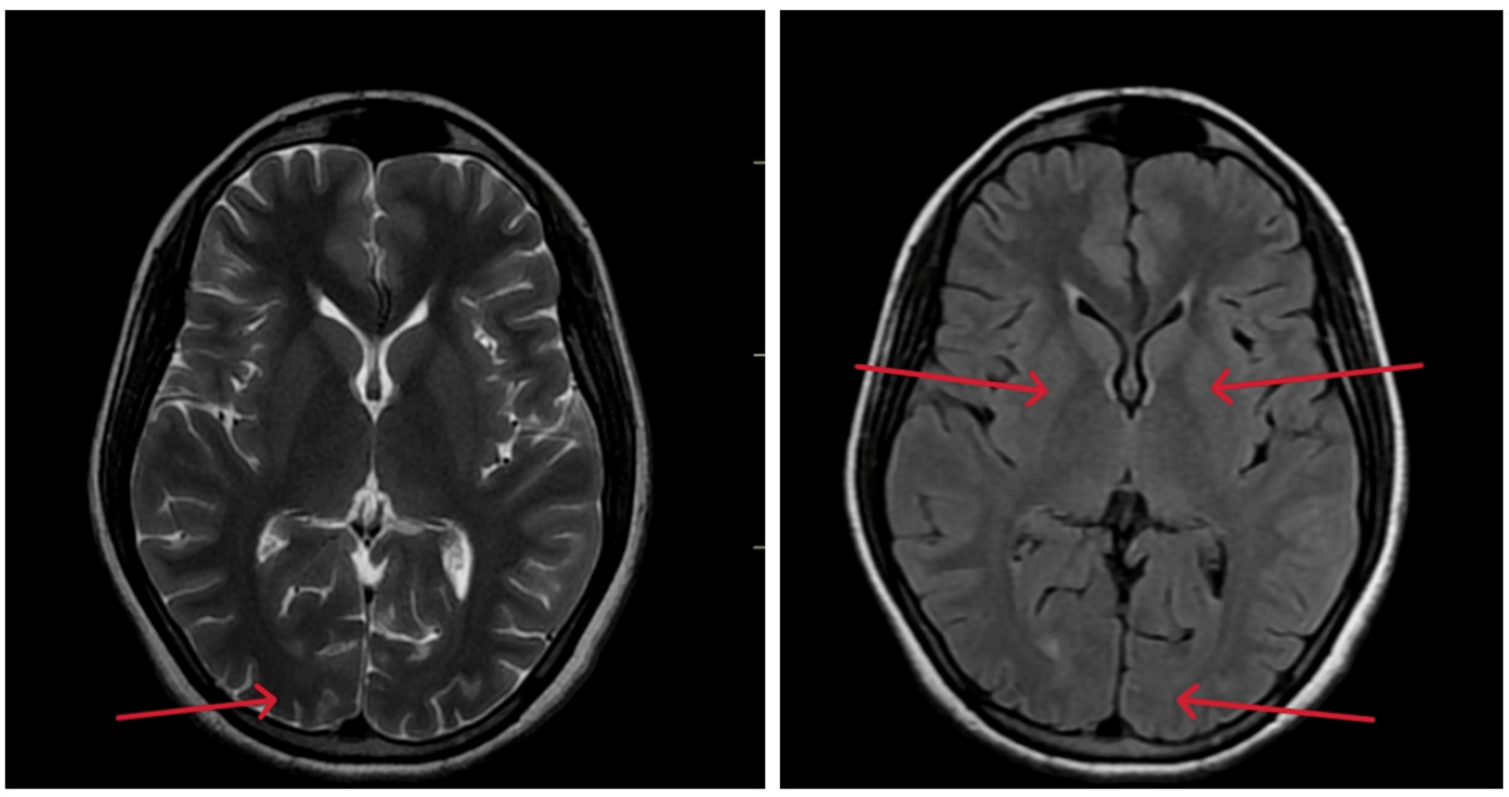
Follow-up MRI of the brain (axial section), five days later, showing significant improvement in the pathology, with no significant lesions observed in the same region as before (red arrow).

The patient was started on anti-epileptic drugs, including Inj. Levetiracetam 2 gm IV stat, followed by 1 gm every 12 hours, injection of low molecular weight heparin for thromboprophylaxis, low-dose diuretics, and other neuroprotective medications. She responded well to the treatment. A follow-up MRI of the brain was performed after five days, showing significant improvement, with no significant lesions observed in the same region as before (Figure 2).

Cerebrospinal fluid analysis was done for the patient and the results are explained in Table 2.

**Table 2:** Cerebrospinal fluid analysis of the patient.

CSF parameter	Result	Normal range
Appearance	Clear	Clear, colorless
Opening pressure	Normal	10-20 cm H₂O
White blood cells	6 cells/µL	0-5 cells/µL
Red blood cells	0 cells/µL	0 cells/µL
Protein	48 mg/dL	15-45 mg/dL
Glucose	65 mg/dL	40-70 mg/dL
Lactate	1.5 mmol/L	1.1-2.4 mmol/L
Oligoclonal bands	Negative	Negative
Gram stain	No organisms seen	No organisms seen
Culture	No growth	No growth

**Figure 3:**
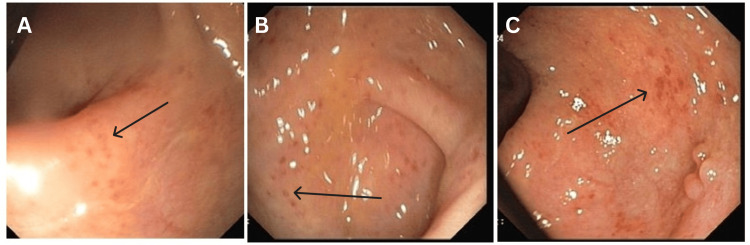
Colonoscopy of the patient showing diffuse areas of inflammation and erosions (black arrows) in the recto-sigmoid portion of the large intestine.

The patient was advised to undergo a colonoscopy after stabilization by the gastroenterologist. The colonoscopy showed proctosigmoiditis, suggestive of UC. Multiple biopsies were taken and sent for histopathological confirmation (Figure 3).

**Figure 4:**
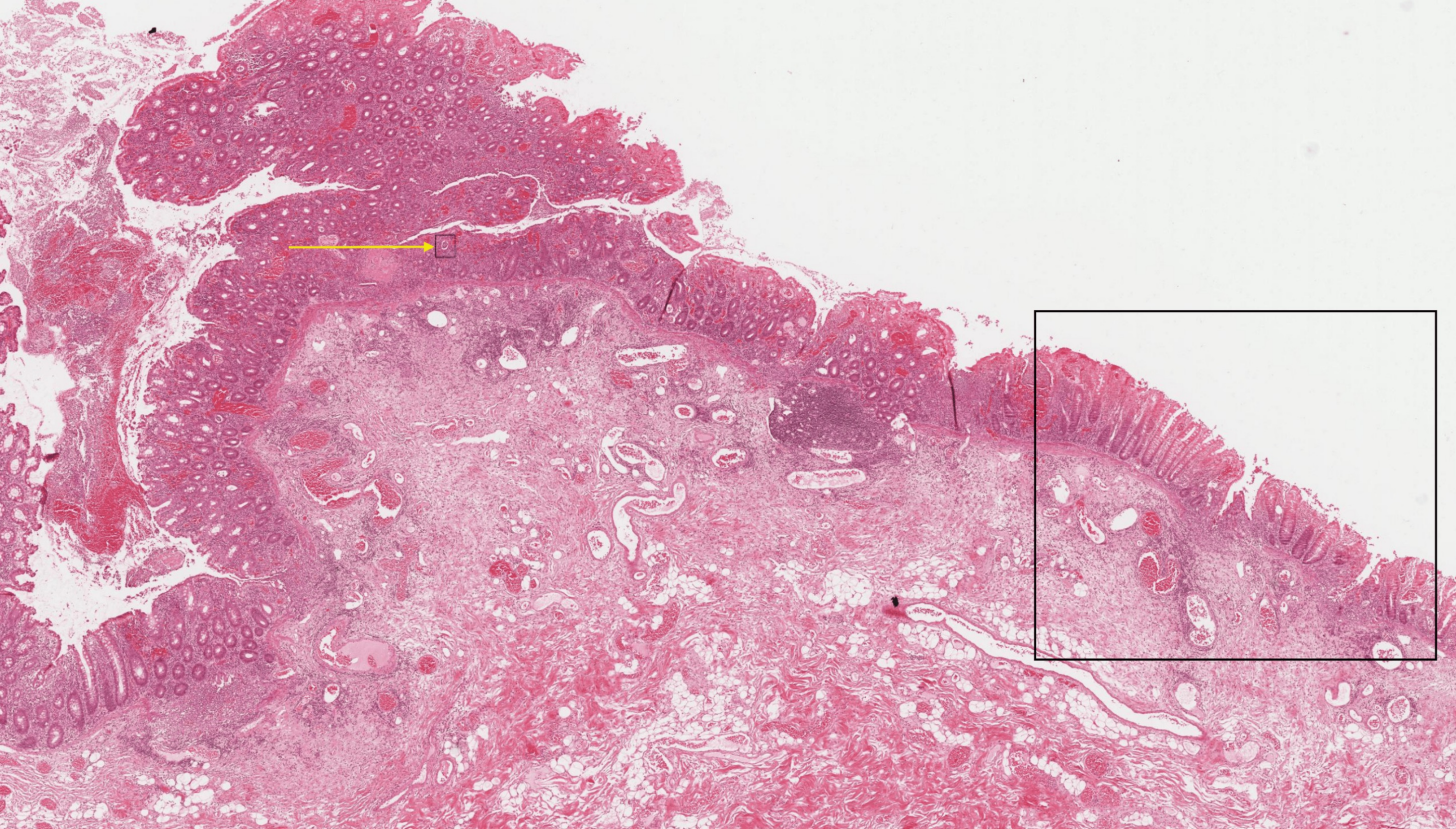
H&E, 4x magnification, section demonstrating chronic active inflammation (crypt abscess - yellow arrow) in the recto-sigmoid, extending proximally in a continuous, diffuse pattern. The changes are limited to the mucosa and superficial submucosa (black box). Histopathological features are suggestive of UC. H&E, hematoxylin and eosin; UC, ulcerative colitis

Histopathological examination confirmed the diagnosis of UC (Figure 4).

After confirming the diagnosis of UC, she was started on tab mesalamine 3.6 g per day, methylprednisolone 1 g per day, and supportive management, which included administering IV fluids, correcting electrolyte imbalances, monitoring and managing blood pressure, and using antiepileptic medications to control seizures. Nutritional support was provided through a digestible diet. Pain management with analgesics and regular neurological assessments were done to monitor neurological status, along with education and psychological support for the patient and family. She was discharged after 12 days of hospital admission.

She returned to the hospital for a follow-up after two weeks. She stated great relief from all symptoms, with occasional loose stools, without any pain or tenderness. She was advised to continue tab mesalamine 3.6 g per day, supportive nutritional supplements, and to return to the hospital if symptoms reappear.

## Discussion

A headache, seizure, altered consciousness, focal neurological impairments, and abnormalities in vision are typical symptoms of PRES, a rare and treatable neurological condition [5]. When a stroke or sudden brain hemorrhage occurs in a small percentage of severe cases, it may be fatal [6].

PRES has been associated with four distinct patterns of imaging: (i) parietal-occipital pattern (22% of cases); (ii) holohemispheric pattern (23% of cases); (iii) superior-frontal pattern (27% of cases); and (iv) mixed expression of the aforementioned patterns (28% of cases) [7].

Frequently developing lesions in the posterior parieto-occipital lobes are consistent with sympathetic innervation, which regulates the vertebrobasilar system's blood flow less efficiently than the carotids [8]. There are now two etiopathogenetic hypotheses have been proposed [2]. In the first, there is an abrupt increase in blood pressure that causes the blood-brain barrier to be disrupted and the autoregulatory systems that regulate cerebral perfusion to fail, both of which result in edema. The correlation between increased blood pressure and PRES lends credibility to this theory. PRES is most commonly associated with high blood pressure (hypertension), which is thought to contribute to the endothelial dysfunction and vasogenic edema characteristic of the condition [8].

Low blood pressure is an unusual finding in PRES, making this case atypical and clinically significant. When PRES presents with low blood pressure, it challenges the traditional understanding of the syndrome's pathophysiology. Several hypotheses can be proposed to explain this rare presentation of low blood pressure, such as systemic inflammation in ulcerative colitis (UC) causing vasodilation due to inflammatory cytokines, medication side effects (e.g., immunosuppressants and biologics), fluid and electrolyte imbalances leading to fluid loss and dehydration, ultimately resulting in hypovolemia, and underlying vascular conditions or cardiac autonomic dysfunctions [9].

According to the second idea, direct toxic effects (cytotoxic, autoimmune, or drug-related) may cause endothelial dysfunction and capillary leakage, which in turn may cause fluid extravasation. Chronic alcoholism, as well as toxins like Methanol consumption being the causative agents for PRES, has been documented in the literature previously [9].

Additional factors that may contribute to this condition include sepsis, autoimmune diseases, acute liver failure, blood transfusions, chronic renal failure, electrolyte imbalance, and human immunodeficiency virus infection [10]. It is probable that endothelial dysfunction or capillary leakage contributes to the presence of proinflammatory cytokines in the blood [2].

The persistent, debilitating disease known as UC is marked by inflammation of the intestinal mucosa. It is a multi-organ system inflammatory condition that is systemic in nature. Less than 5% of instances of UC are linked with central nervous system dysfunction, an uncommon consequence of IBD. These include vasculitis, leukoencephalitis, seizures, and arterial and venous thrombosis [11]. Only 15 case reports of probable cerebral vasculitis in UC have been reported, according to Unnikrishnan et al. Among these reported cases, 11 patients had confirmed vasculitis based on histopathology, angiogram, or serology [12]. Prednisone treatment helped the neurological impairments in nearly all 15 patients to improve. Concurrently, two patients showed improvement when prednisone and cyclophosphamide were taken together, and one instance improved when prednisone, azathioprine, and cyclosporine A were used together. Whether or not UC is involved, the greatest result for PRES appears to come from steroid therapy.

Sporadic instances have been reported where the possible cause of the development of PRES, in our case, can be argued to be cerebral vasculitis. As PRES, as well as cerebral vasculitis, respond well to steroids and anti-epileptic drugs, the patient responded to the treatment.

## Conclusions

There are very few examples of IBD that have been documented in the literature, such as our case of PRES linked to UC. When an IBD patient arrives with neurological symptoms, this relationship should be taken into account. The differential diagnosis is broad, but a well-constructed workup is required. It might be unsettling to see the MRI's severe abnormalities and neurological symptoms. The remarkable recovery of the brain lesions observed in our case, however, indicates that most of these individuals do react favorably to steroid therapy. Hopefully, better diagnostic and treatment parameters may be established with more documentation of this rare association.
